# Supplementary material for: Knowledge of COVID-19 Infection Guidelines among the Dental Health Care Professionals of Jazan Region, Saudi Arabia
Source: Int J Environ Res Public Health. 2022 Feb 11;19(4):2034. doi: 10.3390/ijerph19042034 (PMC8872162; doi:10.3390/ijerph19042034)
Supplement: Supplementary file 1 [file ijerph-19-02034-s001.zip › ijerph-1540434-supplementary.pdf]

Table S1. Questionnaire.

Knowledge of COVID-19 infection prevention and control guidelines before, during, and after performing dental procedures among dental students, interns, and dentists in Saudi Arabia.	
Demographic and general characteristics	Gender
	○ MALE<br>○ FEMALE
	Age
	○ 20-29 YRS<br>○ 30 - 39 YRS<br>○ 40 -49 YRS<br>○ 50 -59 YRS<br>○ ABOVE 60 YRS
	Professional experience
	○ Students in dental clinics<br>○ Intern<br>○ 0-9 yrs<br>○ 10-19 yrs<br>○ 20 yrs and above
	Work/Study sector (Check all that apply)
	○ Academic institute dental clinics<br>○ Private dental clinics<br>○ government hospital dental clinics<br>○ military hospital dental clinics<br>○ Non practising dentists<br>○ others
	Region of study/practice in Saudi Arabia
	○ Riyadh region<br>○ Makkah region<br>○ Madinah region<br>○ Qassim region<br>○ Eastern region<br>○ Asir region<br>○ Tabuk region<br>○ Hail region<br>○ The northern border region<br>○ Jazan region<br>○ Najran region<br>○ Al Baha region<br>○ Al jouf region
	Have you read any guidelines for providing dental services during COVID-19 Pandemic (MOH, WHO, CDC, ADA, NHS, etc)?
	○ YES<br>○ NO
	Have you attended any COVID-19 infection prevention workshops?
	○ YES<br>○ NO
The following questions are relevant to COVID-19 infection prevention "BEFORE" performing dental procedures	1. Patients with non-urgent conditions should be encouraged to maintain proper oral hygiene by consuming a healthy diet, avoiding hard or sticky food, and keeping good oral hygiene practices to preserve their current status.
	○ Strongly Disagree<br>○ Disagree<br>○ Neutral<br>○ Agree<br>○ Strongly Agree
	2. Patients with reversible pulpitis and dentine hypersensitivity should be recommended analgesics if needed, avoid stimuli (cold, hot and acidic drinks or food), apply desensitizing toothpaste regularly to the sensitive area with a finger, and advise the patient to call back if symptoms get worse.
	○ Strongly Disagree<br>○ Disagree<br>○ Neutral<br>○ Agree<br>○ Strongly Agree
	3. Prevent crowding in appointment settings by booking appointments

	o Strongly Disagree<br>o Disagree<br>o Neutral<br>o Agree<br>o Strongly Agree
	4. Any dental procedures should be delayed in patients with a history of COVID-19 for at least a month
	o Strongly Disagree<br>o Disagree<br>o Neutral<br>o Agree<br>o Strongly Agree
	5. High-risk patients like diabetic and immunocompromised patients should be treated early in a dental office opening.
	o Strongly Disagree<br>o Disagree<br>o Neutral<br>o Agree<br>o Strongly Agree
	6. Telephonic triage / Tele dentistry should not be considered an alternative to in-office care.
	o Strongly Disagree<br>o Disagree<br>o Neutral<br>o Agree<br>o Strongly Agree
	7. Patients with fracture / loose tooth fragments or broken restorations should be referred to the designated urgent dental clinics during the COVID-19 pandemic.
	o Strongly Disagree<br>o Disagree<br>o Neutral<br>o Agree<br>o Strongly Agree
	8. The temperature of staff and patients should be monitored daily
	o Strongly Disagree<br>o Disagree<br>o Neutral<br>o Agree<br>o Strongly Agree
	9. Ask dental health care personnel to stay home if they are sick
	o Strongly Disagree<br>o Disagree<br>o Neutral<br>o Agree<br>o Strongly Agree
	10. Patients with fever should be referred to a specific medical centre's treating COVID 19
	o Strongly Disagree<br>o Disagree<br>o Neutral<br>o Agree<br>o Strongly Agree
	11. Accompanying individuals with patients should be allowed in the clinics.
	o Strongly Disagree<br>o Disagree<br>o Neutral<br>o Agree<br>o Strongly Agree
	12. Hand disinfection with 60 - 75% alcohol should be offered upon entrance to the dental office.
	o Strongly Disagree<br>o Disagree<br>o Neutral<br>o Agree<br>o Strongly Agree
	13. Emergency dental care can be provided if a patient's temperature is less than 100.4-degrees Fahrenheit and does not have symptoms consistent with COVID-19.
	o Strongly Disagree<br>o Disagree

	o Neutral<br>o Agree<br>o Strongly Agree
	14. The waiting area should be large with adequate ventilation.
	o Strongly Disagree<br>o Disagree<br>o Neutral<br>o Agree<br>o Strongly Agree
	15. The 2-meter separation between patients is mandatory in waiting rooms and reception areas.
	o Strongly Disagree<br>o Disagree<br>o Neutral<br>o Agree<br>o Strongly Agree
	16. Remove magazines, toys, and other objects which cannot be easily disinfected
	o Strongly Disagree<br>o Disagree<br>o Neutral<br>o Agree<br>o Strongly Agree
	17. Posters in the dental office for instructing patients on standard recommendations for respiratory hygiene/cough etiquette and social distancing should be posted in appropriate places.
	o Strongly Disagree<br>o Disagree<br>o Neutral<br>o Agree<br>o Strongly Agree
	18. It is not required by everyone entering the dental office to use facemasks or cloth face coverings.
	o Strongly Disagree<br>o Disagree<br>o Neutral<br>o Agree<br>o Strongly Agree
	19. Dental procedures require professionals to use Personal protective equipment (surgical caps, gloves, N-95 mask, FFP2 mask, goggles, gowns, and face shields).
	o Strongly Disagree<br>o Disagree<br>o Neutral<br>o Agree<br>o Strongly Agree
	20. It is not required to cover all touchable surfaces with disposable protections.
	o Strongly Disagree<br>o Disagree<br>o Neutral<br>o Agree<br>o Strongly Agree
	21. Patients should not be treated in rooms with negative pressure relative to the surrounding area.
	o Strongly Disagree<br>o Disagree<br>o Neutral<br>o Agree<br>o Strongly Agree
The following questions are relevant to COVID-19 infection prevention "DURING" the dental procedures	22. In case hands are visibly soiled, water and soap should be used at least 20 seconds before using an Alcohol-based hand rub.
	o Strongly Disagree<br>o Disagree<br>o Neutral<br>o Agree<br>o Strongly Agree
	23. Preprocedural mouth rinse like 1.5% hydrogen peroxide or 0.2% povidone should not be used before starting any dental procedure in the patient.
	o Strongly Disagree<br>o Disagree<br>o Neutral

	o Agree<br>o Strongly Agree
	24. Avoid the use of topical spray anaesthesia to prevent gag reflex
	o Strongly Disagree<br>o Disagree<br>o Neutral<br>o Agree<br>o Strongly Agree
	25. Use of rubber dam and N-95 masks are mandatory for aerosol-generating dental procedures
	o Strongly Disagree<br>o Disagree<br>o Neutral<br>o Agree<br>o Strongly Agree
	26. High-volume saliva ejectors can increase aerosol or spatter while performing dental procedures.
	o Strongly Disagree<br>o Disagree<br>o Neutral<br>o Agree<br>o Strongly Agree
	27. Panoramic radiographs or cone-beam computed tomographs should not be used intraoral radiography.
	o Strongly Disagree<br>o Disagree<br>o Neutral<br>o Agree<br>o Strongly Agree
	28. Four-handed dentistry should not be practised for aerosol-generating procedures.
	o Strongly Disagree<br>o Disagree<br>o Neutral<br>o Agree<br>o Strongly Agree
	29. Use of 3-in-1 syringes, air-water syringes, and ultrasonic instruments are allowed for all aerosol-generating dental procedures
	o Strongly Disagree<br>o Disagree<br>o Neutral<br>o Agree<br>o Strongly Agree
	30. Adopt the Atraumatic Restorative Technique and Chemo mechanical caries removal procedure wherever possible
	o Strongly Disagree<br>o Disagree<br>o Neutral<br>o Agree<br>o Strongly Agree
	31. To reduce the clinical time, preferences should be given to bulk-fill composite resin restorations as it permits increments up to 4mm in thickness.
	o Strongly Disagree<br>o Disagree<br>o Neutral<br>o Agree<br>o Strongly Agree
	32. Treatment should be completed in multiple visits wherever possible.
	o Strongly Disagree<br>o Disagree<br>o Neutral<br>o Agree<br>o Strongly Agree
The following questions are relevant to COVID-19 infection prevention "AFTER" performing dental procedures	33. Environmental cleaning and disinfection procedures should be followed after completion of treatment
	o Strongly Disagree<br>o Disagree<br>o Neutral<br>o Agree<br>o Strongly Agree

	34. Clean and disinfect reusable PPE
	○ Strongly Disagree<br>○ Disagree<br>○ Neutral<br>○ Agree<br>○ Strongly Agree
	35. Manage laundry and medical waste following routine procedures
	○ Strongly Disagree<br>○ Disagree<br>○ Neutral<br>○ Agree<br>○ Strongly Agree
